# Enabling multiple intercavity polariton coherences by adding quantum confinement to cavity molecular polaritons

**DOI:** 10.1073/pnas.2206062120

**Published:** 2022-12-27

**Authors:** Zimo Yang, Harsh H. Bhakta, Wei Xiong

**Affiliations:** ^a^Materials Science and Engineering Program, University of California, San Diego, La Jolla, CA 92093; ^b^Department of Chemistry and Biochemistry, University of California, San Diego, La Jolla, CA 92093; ^c^Department of Electrical and Computer Engineering, University of California, San Diego, La Jolla, CA 92093

**Keywords:** polaritons, quantum confinement, coherences, 2D IR, quantum simulation

## Abstract

In this study, the “particle in a box” idea, which was broadly developed in semiconductor quantum dot research, was extended into mid-infrared (IR) cavity modes by applying lateral confinement in an optical cavity. The discrete cavity modes hybridized with molecular vibrational modes, resulting in a quartet of polariton states that can support multiple coherence states in the IR regime. We applied tailored pump pulse sequences to selectively prepare these coherences and verified the multi-coherence existence. The simulation based on Lindblad equation showed that because the quartet of polariton states resided in the same cavity, they were specifically robust toward decoherence caused by fluctuations in space. The multiple robust coherences paved the way for entangled states and coherent interactions between cavity polaritons, which would be critical for advancing polariton-based quantum information technology.

## Significance

A new microcavity infrastructure, with lateral confinements, was designed and built for molecular vibrational polaritons, which are hybrid half-light, half-matter quasiparticles. The newly implemented photonic confinement to the Fabry-Perot cavity created additional “quantized” cavity modes and enabled the formation of a polaritonic multi-qubit systems, also called qudits. This new photonic structure enabled multiple coherences that were robust against environmental fluctuations, addressing a bottleneck in applying molecular polaritons for quantum technology. This work not only served as an important step for developing molecular vibrational polaritons for quantum information technology, such as simulating light harvesting complex, but also had significant implications in realizing topological polariton systems and quantum light spectroscopy for molecular systems.

**Fig. 1.**
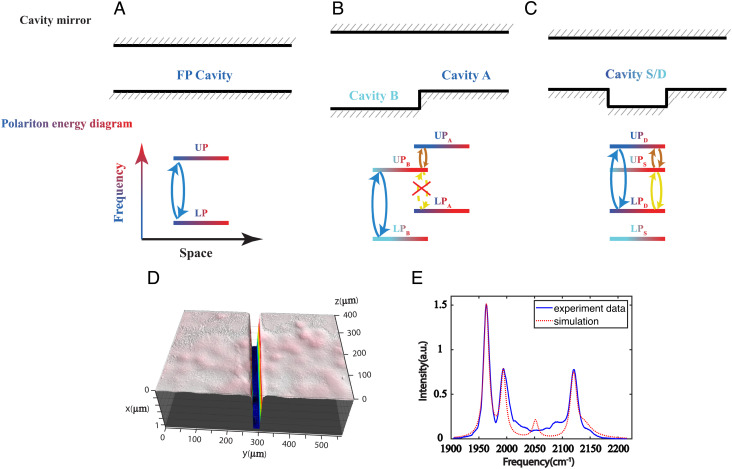
Challenges of creating multiple coherences in cavity polaritons. (*A*) In an FP cavity composed by two flat mirrors, one pair of UP and LP is supported and thereby can only form one coherence |UP〉〈LP| and its conjugate. (*B*) In the dual cavity, two cavity modes are supported due to the longitudinal cavity thickness difference along the lateral dimension. This cavity can support two pairs of UP and LP and enrich the varieties of coherences. However, coherences such as |UP_B_〉〈LP_A_| cannot survive the fluctuations between cavities. (*C*) In this work, we demonstrated the confined cavity by implementing the “particle in a box” concept. In this way, two cavity modes and two pairs of polariton modes are supported in the same spatial location, enabling the creation of coherences among any pairs of polaritons. To clearly show the confinement in the illustration, the vertical dimension was exaggerated. (*D*) A close view of the confined-cavity pattern obtained by optical profilometer. The lateral dimension of the cavity (the short side of the trench) is 25 μm. The depth of the trench is 1 μm. (*E*) The linear transmission spectra obtained by focusing IR beams center at the trenched area on the sample. Two peaks at 1,971 cm^–1^ and 1,995 cm^–1^ are from the confined cavity, whereas the peak at 2,099 cm^–1^ is from the unconfined region. The dashed line is the simulation result.

Molecular vibrational polaritons (1–18) are half-matter, half-light quasiparticles that possess unique abilities to change chemical reactions (3, 10, 12, 15, 17, 19–22), modify energy transfer pathways (7, 14, 23, 24), and have the potential to be an alternative platform for quantum simulation (9, 25–32). When the collective dipole coupling between cavity photon modes and molecular vibrational modes is so strong that the two modes exchange energy at a rate faster than the lifetimes of either mode, the upper and lower polaritons (UP and LP) are formed and the systems reach the so-called vibrational strong coupling (VSC) regime (31–33). Up to now, the majority of molecular vibrational polaritons are formed in Fabry-Perot (FP) cavity, which has one corresponding cavity photon mode for each specific in-plane momentum. These modes at different in-plane momentum form a continuous parabolic dispersion curve. As such, an FP cavity can support only one pair of UP and LP at a specific in-plane momentum, and thereby, have one coherence (30) (i.e., off-diagonal density matrix elements), namely |UP〉〈LP| (or its complex conjugate, Fig. 1*A*). Thus, UP and LP can be treated as one polariton qubit system at ambient conditions (28, 29, 34).

The molecular vibrational polariton-based qubits is a potential platform for quantum simulation with several advantages, such as operating at ambient temperature, ease of tunability of cavities, intrinsic systems for quantum light molecular spectroscopy, and the customizable “designer” vibrational chromophores (35–37). Although similar efforts have been made on exciton polaritons, the single qubit property of the FP cavity has limited the scalability of molecular vibrational polaritons for advancing quantum simulation (34). One way to overcome the limitations is to form multi-qubit systems, also called qudits, using multi-cavity polariton systems. Early work from our group extended the FP cavity into two pairs of polaritons in spatially neighboring cavities (9, 26), which we termed as dual-cavity system herein (Fig. 1*B*). However, the high-frequency coherences composed of polaritons from different cavity modes (referred as intercavity coherences) cannot survive due to decoherence, because polaritons reside in different spatial locations. To address this limitation, a novel cavity structure is needed to multiplex polariton coherences for simulating complex quantum phenomena.

In this work, we overcome the FP cavity limitations and create two cavity modes with distinct energies by applying an orthogonal confinement in FP cavity system. This confinement effect is similar to “particle in a box,” which is widely applied in semiconductor materials (38–45), including quantum dots and wells. Simply put, when the dimensions of a system are close to the wavelength of the particles, only certain wave functions can survive the boundary condition of the spatial confinement, leading to distinct quantum states and tunable energy gaps. However, compared to the confinement effect in semiconductor materials, this phenomenon has not been heavily explored in the IR regime. Here, we implemented confinement to IR cavities to create two photonic modes at a specific in-plane momentum, and we showed that the confined cavity had a discrete dispersion relation with respect to in-plane momentum. We further demonstrated that under VSC conditions, a quadruplet of polaritons (polaritonic qudits) was created which formed coherences between any pairs of polaritons (Fig. 1*C*). Thus, introducing confinement in a single cavity created a foundation for generating qudits with complex coherence states or even entanglements in the future (46–48). This advance could create a potential platform for quantum light spectroscopy and other quantum science and technology (49, 50). Therefore, this was a valuable step for molecular polaritonic quantum information technology.

## Results

### Discrete Dispersion Curve of the Confined Cavity.

To create the confinement effects to the cavity, we fabricated a trench on a distributed Bragg reflector (DBR) optics, and pair it with another flat DBR window to form a cavity (Fig. 1*C*). The trench had a depth of 1 μm, a width of 25 µm, and a length of 7 mm (Fig. 1*D*) (see *Methods* for fabrication details). The two DBR mirrors were then separated by a Teflon spacer of thickness 12 µm to form the cavity, such that the trenched area has a 13 µm cavity thickness (illustrated in Fig. 1*C*. Note: the vertical dimension is drawn to illustrate the confinement effect and is not to scale). The IR transmission spectra of the cavity showed well-resolved peaks at 1,971, 1,995, and 2,099 cm^–1^ (Fig. 1*E*). As the IR laser beam was scanned away from the trench, the doublet peak (1,971 and 1,995 cm^–1^) intensity decreased, and the single peak (2,099 cm^–1^) intensity increased (*SI Appendix*, Fig. S1*B*). Thus, the double peaks were from the trenched area, whereas the isolated peak was from outside of the trenched area.

Considering that the confined-cavity system was formed with bi-planar mirrors, we might think that it also had two cavity modes corresponding to the different cavity longitudinal lengths. However, the appearance of the three peaks could be explained by the “particle in a box” model confined by a finite potential (44, 51, 52), by considering the confinement in the orthogonal direction in the trenched area. Under the condition of $k_{\|}\;\ll \;k_{⊥}$, cavity modes have energy dispersion relationship described as

[1] $$E_{cav}\;=\;E_{cav}(k_{\|}=0)+\frac{\hbar^{2}{k_{\|}}^{2}}{2m_{\mathrm{cav}}},$$

[2] $$E_{\mathrm{cav}}(k_{\|}=0)\;=\;\frac{\hbar c}{n_{c}}k_{⊥},$$

where $E_{\mathrm{cav}}$ is the energy of the cavity modes, $k_{\|}=\frac{\pi n}{L_{\|}}$ is the in-plane momentum, $k_{⊥}=\frac{\pi n}{L_{⊥}}$ is the normal momentum, ℏ is the reduced Planck’s constant, mcav = Ecav(k‖=0)c2nc2 is the effective mass of the cavity, $n_{c}$ is refractive index of the media inside the cavity, $L_{\|}$ and $L_{⊥}$ are the width and height of the cavity, and n is an integer (31).

**Fig. 2.**
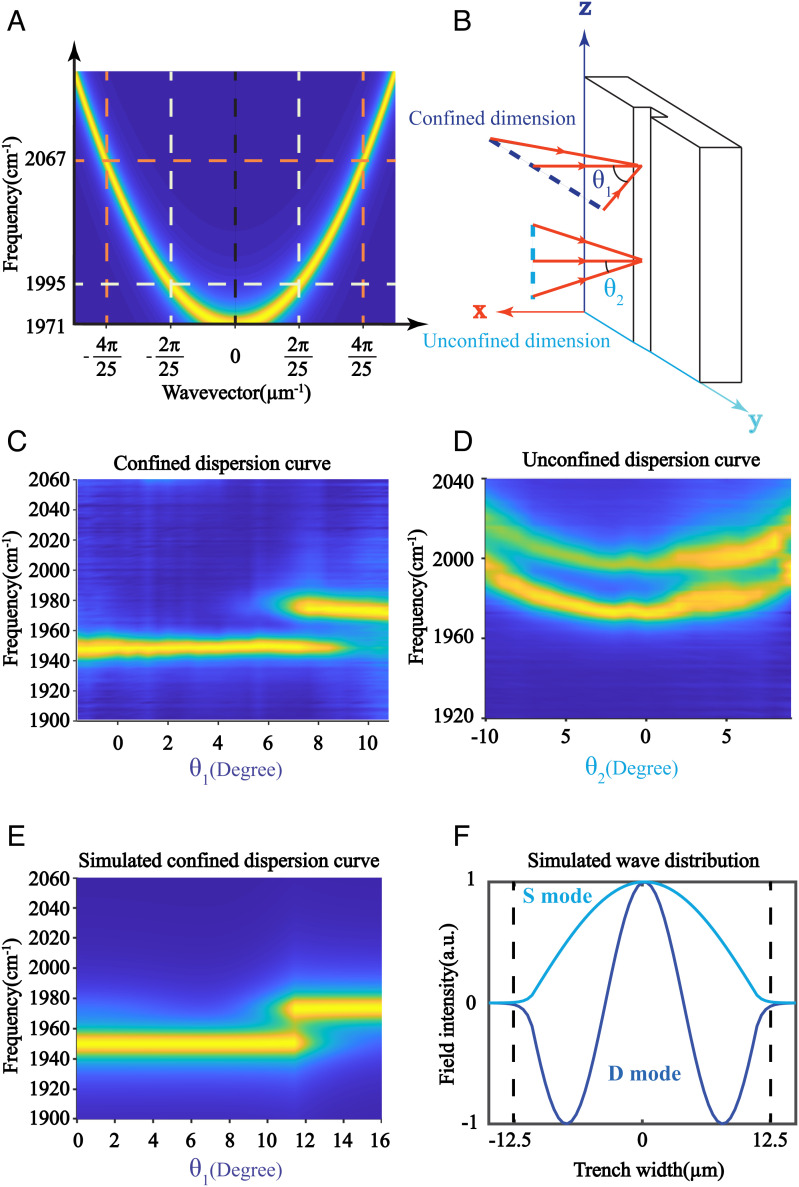
Dispersion curves and EM wave distributions of the confined-cavity modes. (*A*) The dispersion curve of a free particle. When confined in a box, the particles only allow discrete momentum, labeled by vertical dashed lines, allowing only modes with specific momentums to be created (indicated by cross points of dashed lines of the same colors). (*B*) Illustration of scanning the incidence angles along confined (θ_1_) and unconfined (θ_2_) dimensions. The corresponding normalized experimental dispersion curves along (*C*) the confined dimension and (*D*) the unconfined dimension agree with the particle in a box model: Along the confined dimension, the modes are discrete, whereas the unconfined dimension has continuous dispersion curves. (*E*) The simulated dispersion curve along the dimension of confinement. (*F*) The simulated E-field spatial distribution of cavity modes S and D. The D mode has more nodes, but both modes reside in the same region.

When the orthogonal dimension is unconfined, like in the FP cavity, $L_{\|}$ is infinite and $k_{\|}$ is continuously defined. Thus, the dispersion curve is continuous (Fig. 2*A*). However, when $L_{\|}$ is finite and on the order of λ, the wavevector $k_{\|}$ changes discretely with step-size of $\pm \frac{\pi }{L_{\|}}$, due to the interferences imposed to meet the boundary conditions. In the trenched cavity, when $L_{\|}$ = 25 µm, the even modes are observable with $k_{\|}$ being $0,\pm \frac{2\pi }{25},$ and $\pm \frac{4\pi }{25}$, beyond which, the corresponding cavity energy would be above the energy of an unconfined cavity (Fig. 2*A*). On the other hand, the odd modes could not be resolved well under current experimental conditions (*SI Appendix, Experimental Data for More Confined Cavities*). Therefore, the two trenched area peaks in the linear spectra should correspond to the two lowest energy even modes resulting from the orthogonal confinement of electromagnetic (EM) waves inside the trenches. From Eq. **1** and **2**, the energy separation between cavity modes depends on the trench width ($L_{\|}$) and depth ($L_{⊥}$) and refractive index of the media inside of the cavity (n_c_, through the effective mass). We verified the “particle in a box” model by modifying $L_{\|}$ and n_c_ and showed that the experimental peak separation agreed well with the model prediction (*SI Appendix*, *Experimental Data for More Confined Cavities*). We also found that the optimal condition for resolving the polariton coherence experimentally was a trench with a width of 25 µm and depth of 1 µm and a cavity of thickness 12 µm, which would be the cavity parameters used in experiments below.

The discrete dispersion, a character for the “particle in a box” cavity modes, was experimentally confirmed by scanning the linear spectra of the trenches in the confined and unconfined dimensions (Fig. 2*B*). When the beam incidence angle was adjusted in the plane perpendicular to the long axis of the trench (confined dimension, θ_1_), the dispersion curve showed discrete states. Specifically, the frequency of the cavity mode did not change as a function of incident angle (or in-plane momentum), but the relative intensity of the states did. Within a certain angle range, two cavity modes could be resolved well (Fig. 2*C*). In contrast, when the incidence angle was tuned in a plane parallel to the long axis of the trench (unconfined dimension, θ_2_), two continuous dispersion curves of a classical FP cavity were obtained (Fig. 2*D*), because of the two discrete states that resulted from confinements in the other dimension.

The “particle in a box” model qualitatively described the cavity mode characteristics and provides intuition for understanding the system. A more complete description of the system was obtained by solving the Maxwell’s equations (26) in two dimensions, with the geometry of the cavity mirrors as the boundary conditions (*SI Appendix*, *Simulation of Cavity Modes Linear Spectrum*). The frequencies of cavity photon modes and their respective EM field distributions were obtained as the solution of the Maxwell’s equations. They were subsequently used to calculate linear spectra, where the intensities of cavity modes were determined by the convolution of the corresponding EM field distributions and the Gaussian laser beam profile. The calculated spectrum reproduced the experimental measurement well (Fig. 1*E*), so did the calculated discrete dispersion curve (Fig. 2*E*) and the spatial dependence of the peak intensities (*SI Appendix*, Fig. S1*C*). We noted the confined-cavity mode could also be described similar to Gaussian Transverse electromagnetic (TEM) mode of FP cavity with spherical mirrors but with different boundary conditions (53).

The additional information from the simulation was the EM field intensity distribution (Fig. 2*F*) for the two trenched-cavity modes (1,971 and 1,995 cm^–1^). The lowest energy bright mode (S mode) had zero node, whereas the next “visible” mode (D mode) had two nodes. The mode with one node (P mode) was not visible experimentally and from simulation (Fig. 1*E*). Its invisibility was a net result of a convolution of the mode field distribution with the Gaussian beam profile. To see the P mode, it required a tightly focused IR beam (~20 µm) that were narrower than the mode spatial distribution and other strenuous conditions (see details in *SI Appendix*, *Experimental Data for More Confined Cavities*), which were beyond our experimental limit. Because the two modes (S and D) were eigenvectors (EM field distributions), they were orthogonal with and should not interact with each other. However, later we would show that when polaritons were formed among them, they would interact nonlinearly by sharing the matter components in the same spatial area.

### Quadruplet Polaritons in Confined Cavities and Their Nonlinear Interactions.

**Fig. 3.**
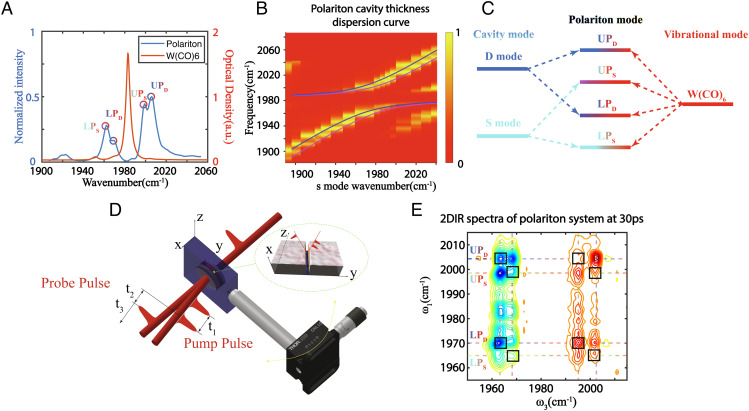
Confined polaritons and the interactions between polaritons. (*A*) Absorpsion spectrum of W(CO)_6_ and transmission linear spectrum of polaritons with confined-cavity modes around zero detuning. Four peaks are observed for four polariton modes. (*B*) Dispersion curve of the confined polariton system and calculated polariton mode dispersion curve using the 4 × 4 Hamiltonian (solid line). (*C*) Energy diagram of confined polariton modes summarizing the 4 × 4 Hamiltonian. S and D modes hybridize with the vibrational modes of W(CO)_6_, respectively, to form two pairs of polariton states. The cavity and vibrational modes are color coded, and the gradient color of polariton modes suggests their approximate compositions (blue for D mode, cyan for S mode, red for molecular vibrational modes). (*D*) Illustration of the 2D IR pulse sequence, geometry used to vary the angle of incidence. *Inset*: The incident angle was varied by the goniometer along the confined dimension (y). (*E*) 2D IR spectra of polariton in the confined cavity at t_2_ = 30 ps. The cross-peaks (highlighted in boxes) indicate nonlinear interactions between polariton modes.

The double modes in the confined cavity presented an opportunity to create multiple polariton states in one cavity. To achieve this, we filled the system with saturated W(CO)_6_ in hexane (red curve in Fig. 3*A* for the spectrum) to form polaritons. Because the cavity modes were discrete in momentum space, we measured the dispersion curve by changing the cavity thickness instead of angle. Near zero detuning, VSC led to four polaritons (Fig. 3*A*), named UP_D_, UP_S_, LP_D_, and LP_S_, from high frequency to low frequency. When away from zero detuning, some polaritons could not be well-resolved from each other because those modes are too close in frequency, and therefore, the systems appeared to have three peaks (Fig. 3*B*). This system was modeled by a 4 × 4 Hamiltonian, in which two cavity modes coupled to two subensembles of vibrational modes separately (*SI Appendix*, *Modeling of the Cavity Thickness Dispersion Curve with 4 × 4 Hamiltonian Matrix*). The modeled dispersion curve (solid lines in Fig. 3*B*) followed the experimental trend well, giving a collective coupling strength of 21 cm^–1^ for both cavity modes S and D, respectively. Thus, VSC in the confined cavity led to a quadruplet polariton system (Fig. 3*C*). The decoupling between the two cavity modes was not completely surprising, because the two modes were orthogonal to each other (54, 55).

However, it was still possible that the molecular vibrational modes strongly coupled to one cavity mode and, at the same time, weakly coupled to the other mode. This type of coupling scheme could facilitate the nonlinear interactions between polaritons. We demonstrated this point by conducting 2D IR spectroscopy of this quadruplet polariton system. In 2D IR, an IR pulse excites the systems into a coherence, oscillating during t_1_, which is transferred by the second IR pulse to a coherence or population state, evolving in t_2_. Then, a third IR pulse transfers the systems into another coherence, resulting in a macroscopic polarization that emits an IR signal (4, 30, 56–59) (Fig. 3*D*). Thus, 2D IR can reveal nonlinear interactions between coherences created by the pulses, which appear as cross-peaks.

At t_2_ = 30 ps, the 2D IR spectra resolved all four polariton modes along the diagonal and showed cross-peaks among all polaritons (Fig. 3*E*). These cross-peaks were either due to Rabi splitting contraction or absorptive features from excited state absorption of dark states, as described in previous works by us and Owrutsky et al. (4, 16, 27). In brief, at t_2_ = 30 ps, excited polaritons already relaxed to the first excited state of the dark modes, which reduced the concentration of molecular absorbers being strongly coupled to the cavity modes, and caused a decrease of the Rabi splitting. The contraction of Rabi splitting led to the derivative spectral features on the ω_3_ = ω_UP_ region; on the ω_3_ = ω_LP_ side, the Rabi splitting contraction was overwhelmed by the absorption of the first excited state of dark modes, due to its anharmonicity (4, 16, 60). Thus, similar to what we learned from the dual-cavity polariton systems (26), these cross-peaks were caused by the polaritons sharing the same dark modes, indicating all polaritons were interacting with each other at long time delay (e.g., 30 ps).

### Preparing Arbitrary Polariton Coherences and Comparison with the Dual-Cavity Polaritons.

**Fig. 4.**
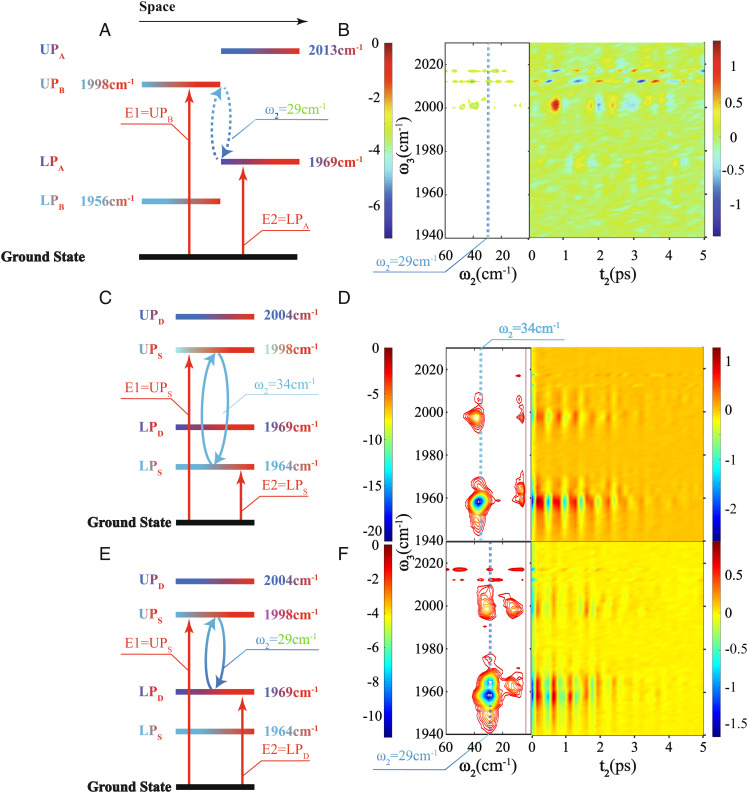
Energy diagrams of coherence in dual and confined-cavity polariton systems and the corresponding coherence signals. Coherence between UP_B_ and LP_A_ (*A*) originated from the different cavity modes that are spatially separated is not observed in (*B*), while the coherence between UP_S_ and LP_S_ (*C*) originated from the same cavity modes of the confined cavity shows strong coherence in (*D*). In (*E*), the coherence is between UP_S_ and LP_D_. While the composing polaritons are from different cavity modes, they still reside in the same physical locations. As a result, it also shows strong coherence signals in (*F*). Regardless of the coherences which are from the same or different cavity modes, they can be created in the confined-cavity systems, and decoherence is on the same timescales. This comparison suggests that the intercavity polariton coherences are more robust against fluctuations in confined cavities than the ones in dual cavities.

Next, because the polaritons in the confined cavity had strong nonlinear interactions, we examined whether the coherences in the confined cavity could be more robust to fluctuations than the ones in a dual-cavity system. In the dual-cavity polariton system, as shown in Fig. 4*A*, if the coherences were created from polaritons residing in two spatially separate cavities, and the coherence frequency was large (e.g., 30 cm^–1^), the coherences could not survive environmental fluctuations (26). This was shown in Fig. 4*B*, where there was no clear coherence oscillation in the time domain, and the corresponding Fourier transform along t_2_ resulted in unclear peaks. In the confined-cavity polaritons, all polariton states were in the same cavity physically, which could possibly lead to more robust coherences.

To examine whether any arbitrary coherence could be created in confined-cavity polaritons, we tailored the first two IR pulses of 2D IR using a mid-IR pulse shaper (PhaseTech Spectroscopy, Inc.) to create targeted coherences. For example, we shaped the first pump pulse to be centered at $\omega_{{\mathrm{UP}}_{S}}$ to excite the system to ${|UP}_{S}\rangle \langle 0|$ and the second pulse to be centered at $\omega_{{\mathrm{LP}}_{s}}$ to initiate the coherence ${|UP}_{S}{\rangle \langle LP}_{S}|$ (Fig. 4*C*) (25, 61). To characterize this coherence, we then took the corresponding 2D IR spectra while scanning $t_{2}$. The 2D IR signal oscillated at the frequency of $\omega_{{\mathrm{UP}}_{S}}-\omega_{{\mathrm{LP}}_{S}}$ during t_2_ (Fig. 4 *D*, *Right*), indicating the coherence ${|UP}_{S}{\rangle \langle LP}_{S}|$ is prepared. To better visualize the coherence, we Fourier transformed the 2D IR spectra along the $t_{2}$ axis, to plot 2D spectra of $\omega_{2}$ and $\omega_{3}$ at specific $\omega_{1}$ (62). For example, when coherence ${|UP}_{S}{\rangle \langle LP}_{S}|$ was prepared, the spectra cut at $\omega_{1}=\omega_{{\mathrm{UP}}_{S}}$ (Fig. 4 *D*, *Left*) clearly showed a peak of 34 ${\mathrm{cm}}^{-1}$ along $\omega_{2}$ axis, agreeing with $\omega_{{\mathrm{UP}}_{S}}-\omega_{{\mathrm{LP}}_{S}}=34\;{\mathrm{cm}}^{-1}$ and demonstrating that this coherence existed.

While it was not surprising to prepare coherences between polaritons from the same cavity modes, referred to as intracavity coherences, the real challenge was to prepare coherences between polaritons from different cavity modes, such as S and D modes, e.g., intercavity coherences. It was a challenge because these polaritons could be subjected to different fluctuations (26), and it was also where the dual-cavity polaritons failed to achieve coherence. As shown in Fig. 4 *E* and *F*, coherence such as |${\mathrm{UP}}_{S}$〉〈${\mathrm{LP}}_{D}$| could, indeed, be prepared and were robust against environmental fluctuations, as indicated by the peak at $\omega_{2}=29{\;\mathrm{cm}}^{-1}$. Similarly, coherences of |${\mathrm{UP}}_{D}$〉〈${\mathrm{LP}}_{D}$| and |${\mathrm{UP}}_{D}$〉〈${\mathrm{LP}}_{S}$| could be prepared (*SI Appendix*, Fig. S5). The highest frequency of these intercavity mode coherences was 40 cm^–1^. This was in sharp contrast to the dual-cavity systems, which did not support intercavity coherences beyond 10 cm^–1^, limiting coherences that could be created (26) (Fig. 4 *A* and *B*). We note that there were also peaks at lower ω_2_ frequency, which could be due to coherence transfer (63, 64), a topic beyond the scope of the current work.

### Origin of Coherence Robustness.

Although energy fluctuations due to thermal-activated solvent motions acted as a source of decoherence in both the confined-cavity and dual-cavity systems, the difference between them was that the polaritons in the dual cavity resided in two different physical locations, whereas all polaritons were located in the same physical location in the confined cavity. Thus, we hypothesized that the origin of the more robust coherences in the confined cavity versus the dual cavity was the lack of solvent fluctuations between cavities in different spaces (in short, spatial fluctuations). As a result, the intercavity coherence of the dual-cavity polariton suffered both energy and spatial fluctuations, making it difficult to create.

To model the decoherence, we simulated the coherence signal using Lindblad equation:

[3] $$\begin{array}{c} i\partial_{t}\rho =L\left[\rho \right]\equiv \left[H,\rho \right]+i\sum_{a}\left(F_{a}\rho F_{a}^{†}-\frac{1}{2}\left\{F_{a}^{†}F_{a},\rho \right\}\right), \end{array}$$

where ρ was the time-dependent density matrix, H was the Hamiltonian of the system, and *F*_*a *_was the Lindblad operators phenomenologically indicating different sources of decoherence. For the polaritons in both cavity systems, the ubiquitous source of decoherence was from energy measurement, described as $\begin{array}{c} F_{1}=\gamma_{1}H=\gamma_{1}\left(\begin{array}{cccc} E_{UP_{B}} & 0 & 0 & 0\\ 0 & E_{UP_{A}} & 0 & 0\\ 0 & 0 & E_{LP_{B}} & 0\\ 0 & 0 & 0 & E_{LP_{A}} \end{array}\right) \end{array}$, where *E_i_* was the eigen energy of polariton *i*. In the dual-cavity polariton, an additional source of decoherence was the spatial measurement, $F_{2}=\gamma_{2}\left(\begin{array}{cccc} x_{B} & 0 & 0 & 0\\ 0 & x_{A} & 0 & 0\\ 0 & 0 & x_{B} & 0\\ 0 & 0 & 0 & x_{A} \end{array}\right)$, where *x_j_* was the position of cavity *j*.

We analytically solved the Lindblad equation and found that polariton coherences could be summarized in two categories: for coherence from same cavity region, such as ${|UP}_{A}\rangle \langle {\mathrm{LP}}_{A}|=e^{(i(E_{UP_{A}}-E_{LP_{A}})-{\gamma_{1}}^{2}{{(E}_{{\mathrm{UP}}_{A}}-E_{{\mathrm{LP}}_{A}})}^{2})t}\;$, which only had decoherence from energy fluctuations; and for the coherence from different cavity regions, such as ${|UP}_{A}\rangle \langle {\mathrm{LP}}_{B}|=e^{(i(E_{UP_{A}}-E_{LP_{B}})-{\gamma_{1}}^{2}{{(E}_{{\mathrm{UP}}_{A}}-E_{{\mathrm{LP}}_{B}})}^{2}-{\gamma_{2}}^{2}{{(x}_{A}-x_{B})}^{2})t}$, which had decoherence from both energy and spatial fluctuations. The analytical results agreed with our intuitions.

**Fig. 5.**
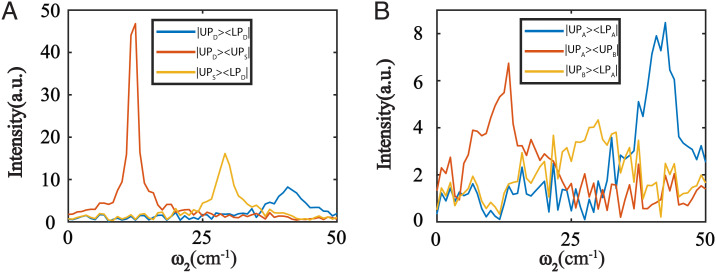
Simulation of decoherence in confined- and dual-cavity systems. (*A* and *B*) are corresponding simulation results of confined cavity (Fig. 1*C*) and dual cavity (Fig. 1*B*). The largest difference is that the intercavity mode coherence |UP_S_〉〈LP_D_| in (*A*) is stronger than coherence |UP_B_〉〈LP_A_| in (*B*). This result suggests that the phase fluctuations experienced by polaritons residing in different locations accelerate decoherences. Thus, coherence is more robust in confined cavities than in dual cavities.

The numerical simulation based on the equations above suggested that the polariton coherences in the confined cavity were more robust to decoherence than coherences in the dual-cavity system. While coherences with larger frequencies had smaller amplitudes (i.e., faster decoherence) in the confined-cavity system (Fig. 5*A*), coherences from any polariton combination could be created—even if coherences were prepared from polaritons from different cavity modes. In contrast, the intracavity coherences (${|UP}_{A}\rangle \langle {\mathrm{LP}}_{A}|$) in the dual-cavity system (Fig. 5*B*) remained strong because of lack of spatial-led decoherence. However, the intercavity coherences ${|UP}_{B}\rangle \langle {\mathrm{LP}}_{A}|$ deteriorated significantly, becoming barely distinguishable from the noise level due to the joint effects of energy and spatial-led decoherences. We emphasize that although the intercavity coherence ${|UP}_{B}\rangle \langle {\mathrm{LP}}_{A}|$ had smaller coherence frequency than ${|UP}_{A}\rangle \langle {\mathrm{LP}}_{A}|$, the overall decoherence was still faster because of the extra spatial fluctuation. However, for certain intercavity coherences, such as ${|UP}_{A}\rangle \langle {\mathrm{UP}}_{B}|$, decoherence due to energy fluctuations was slow because of the small coherence frequency. Therefore, only spatial fluctuation significantly contributes to decoherence, and this coherence peak could still be measured (Fig. 5*B*). Thus, this simulation indicated that the lack of spatial fluctuations mitigated the decoherences of confined-cavity polaritons comparing to those of dual-cavity polaritons. More advanced theory would need to be developed to quantitatively model the decoherence which was out of the scope of the current work.

## Discussion

Using the concept of “particle in a box,” we realized multi-cavity modes by applying confinement to the lateral dimension of a trenched geometry cavity. The multiple cavity modes were simulated by solving Maxwell’s equations. By VSC, the confined cavity supported a quadruplet polariton qudit system with multiple coherences. We showed that, unlike previously reported dual cavities, confined-cavity polaritons could prepare multiple coherences that were robust to decoherence from energy and spatial fluctuations. Based on Lindblad dynamics (65), we showed that the tolerance to environments was because polaritons in confined cavities did not suffer from spatial fluctuation the way polaritons did in a dual-cavity. With deeper trenches, we expect to see more resolved localized modes mimicking atoms but with a small effective mass, thereby achieving phenomena that are difficult to achieve by atoms or molecules in nature. Furthermore, as “particle in a box” is the most basic concept in semiconductor nanomaterials, its implementation to cavity polaritons warrants more investigation on integrating existing advanced nanomaterial designing principles to polaritonics.

The presented quadruplet polariton states and associated coherences in the confined cavity delivered new opportunities for polariton coherences. Hosting multiple coherences in the same cavity region could lead to entanglements. If two of the confined cavities are brought together with a properly designed potential barrier, it would be possible for the D state polaritons to form bonding and antibonding orbitals, whereas the S states remain at the core level. This design could simulate molecules or other coupled systems, such as light harvesting complexes (66), and realize topological systems (67, 68) for future investigations. One barrier toward quantum simulation is the fast decoherence, which could be further alleviated by lower the system temperature (but still much higher than cryotemperature) to freeze solvent motions. The confined-cavity polaritons also provide new platforms for realizing cavity-based entangled photon sources and entangled photon spectroscopies (49, 50). Furthermore, in the polariton chemistry community, it was reported that polariton-modified chemical reaction depends on the detuning, i.e., the energy differences between vibrational modes and cavity modes at zero in-plane momentum (19, 69). However, comprehensive understanding of detuning dependence is complicated by the continuous cavity modes, because many modes at nonzero in-plane momentum can also couple to the molecular modes. Yet, cavity modes at zero in-plane momentum appeared to have greater influence on polariton chemistry than those other modes. The discrete dispersion curve of the confined cavity provides a unique opportunity in resolving this question, because a single discrete cavity mode can be designed such that molecular vibrations essentially only couple to it and not to other modes at nonzero in-plane momentum. Thus, the confined cavity could avoid complications caused by coupling to the continuous cavity modes at higher momentum and provide a clear system for understanding detuning dependence in polariton chemistry.

## Materials and Methods

### Confined-Cavity Fabrications.

To generate confined-cavity mode, two cavity mirrors were needed. One mirror is a flat CaF_2_ window with 92% reflectivity DBR dielectric coating (Thin Film Corp.) around 5 µm wavelength. For the other mirror, a trench was designed using AutoCAD, where the width and length were specified. The cavity mirror with a trench was then fabricated on a CaF_2_ substrate through a lithography process. We first spin coated the negative photoresist on substrate and exposed designed trench area with 375 nm laser (Heidelberg MLA150). Then we used developer solution RD-6 to remove the photoresist at all but the trenched area. The entire optics was then deposited with 1 µm ZnO (the depth of the trench) using Denton Discovery 18 Sputter system. Later, the ZnO on photoresist at the trenched area was lifted off using acetone. Thus, all areas were covered by 1 µm ZnO layer, except the lifted off area, forming the trench geometry. Then, the DBR material was deposited on the top. The DBR layers were four pairs of alternative layers of Ge and ZnO with thickness of 420 nm and 340 nm, respectively. The resulted DBR mirror had a reflectivity of 96% around 5 µm. Two mirrors were put into a demountable liquid cell (Harrick Scientific) and separated by a 12 µm Teflon spacer to form the confined-cavity mode.

### 2D IR Spectroscopy.

2D IR spectroscopy was applied to investigate the light–matter interaction of a confined polariton system (more detailed 2D IR setup and data acquisition are described in *SI Appendix*, *2D IR Spectrometer for Microcavity System*). A 800-nm laser pulses (~35 fs, ~5 W, 1 kHz) generated by an ultrafast Ti:Sapphire regenerative amplifier (Astrella, Coherent) were sent into an optical parametric amplifier (OPA) (TOPAS, Light Conversion) which outputs tunable frequency near-IR pulses. Then the mid-IR beams are temporarily and spatially overlapped on a DFG crystal (a type II AgGaS2 crystal, Eksma) to generate mid-IR around 5 μm. A CaF_2_ wedge separates the beam into pump (95% power) and probe (5% power) parts. The pump beam was sent into a Ge-Acoustic Optical Modulator-based mid-IR pulse shaper (QuickShape, PhaseTech) and was shaped into double pulses with tunable time separation t_1_. The double pump pulses and a probe pulse were arranged in a pump probe geometry to conduct 2D IR measurements. The coherent vibrational states were generated during t_1_ and t_3_, respectively. The first coherence was characterized by scanning t_1_, whereas the second coherence during t_3_ created a macroscopic polarization that emitted IR signals. The IR pump probe signals were directly measured along ω_3_ (probe frequency) at specific t_1_ and t_2_ using the spectrograph and the MCT detector (PhaseTech). A series of pump probe spectra at various t_1_ was Fourier transformed to the frequency domain as ω_1_ (pump frequency) and to obtain the 2D IR spectra. Delay time between the second pump and probe pulses, t_2_, was scanned, using a motorized translation stage, to characterize population or coherence dynamics. All data were collected using a home-written LabView program.

### EM Simulation.

 The wave equation for the photon mode on the cavity lattice was solved by discretizing the Laplacian operator on a 1 × 400 grid in a unit cell along the confinement dimension. This could be treated as a one-dimension problem because the length of the trench was around 7 mm, which could be seen as infinity when compared to the diameter of the IR beam (around 50 µm). After finding the eigen photon modes, the S-wave mode and D-wave mode were selected as cavity modes. The intensity of cavity modes was calculated by the convolution between the corresponding eigen function and the Gaussian beam profile with a size of 50 µm. Refer to *SI Appendix* for more details.

## Supplementary Material

Appendix 01 (PDF)

## Data Availability

All study data are included in the article and/or *SI Appendix*, and related data has been uploaded to https://doi.org/10.5281/zenodo.7426179.
