# Supplementary material for: Enabling multiple intercavity polariton coherences by adding quantum confinement to cavity molecular polaritons
Source: Proc Natl Acad Sci U S A. 2022 Dec 27;120(1):e2206062120. doi: 10.1073/pnas.2206062120 (PMC9910592; doi:10.1073/pnas.2206062120)
Supplement: Supplementary file 1 — Appendix 01 (PDF) [file pnas.2206062120.sapp.pdf]

PNAS

Supporting Information for

Enabling Multiple Intercavity Polariton Coherences by Adding Quantum Confinement to Cavity Molecular Polaritons

Zimo Yang, Harsh H. Bhakta, Wei Xiong

Wei Xiong

Email: w2xiong@ucsd.edu

This PDF file includes:

- Supporting text
- Figures S1 to S5
- SI References

Supporting Information Text

1.Simulation of cavity modes linear spectrum

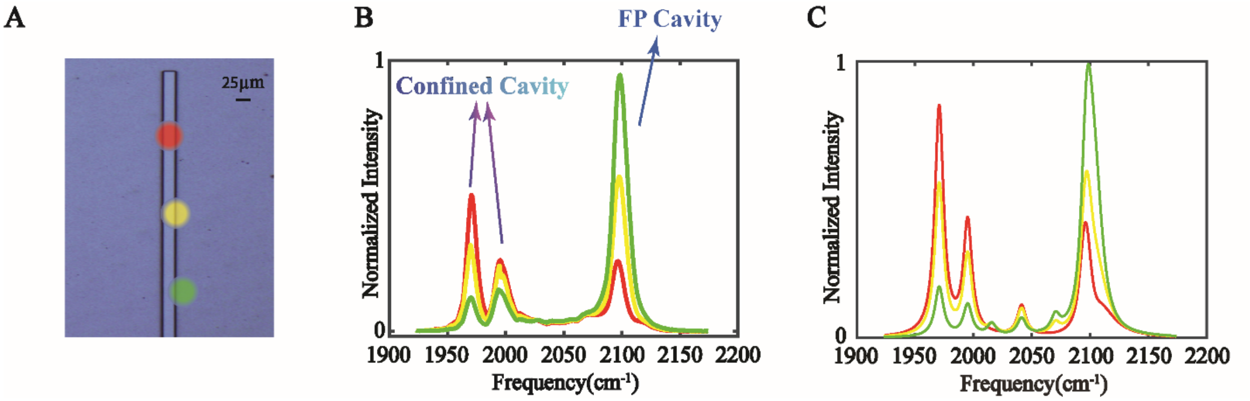

Figure S1 (A) The optical microscope image of the Ge/ZnO DBR window that composes the confined cavity. (B) experimental and (C) simulated linear spectra at different IR beam positions. The relation between linear spectra and corresponding beam positions is indicated by the color in (A).

The photon mode could be described by the wave equation of electric field $(-c_{\parallel}^2\partial_{\parallel}^2 + c_{\perp}^2\left(\frac{\pi}{\mathrm{d}(r)}\right)^2)\boldsymbol{E}(r) = \omega^2\boldsymbol{E}(r)$ .d(r) is the cavity thickness at position r considering the lowest cavity confinement in z-dimension (surface normal). $c_{\parallel}$ and $c_{\perp}$ are the effective velocity in in-plane and perpendicular directions. Then it could be discretized on a 1x400 grid as $(\frac{c_{\parallel}^2}{\delta^2}(2\delta_{i,j} - \delta_{i,j+\hat{x}} - \delta_{i,j-\hat{x}}) + c_{\perp}^2(\frac{\pi}{d_j})^2\delta_{i,j})\boldsymbol{E}_j = \omega^2\boldsymbol{E}_i$ and solved by diagonalization with an open boundary condition. The eigenvalues are cavity modes and eigenvectors are corresponding electric field distributions. The IR beam is represented by a Gaussian of which the center corresponds to the IR focused position and its imaginary part corresponding to the angle of the IR beam. The peak intensity is calculated by the convolution of cavity mode electric field spatial distribution and beam profile. By changing the center of the beam (Fig.S1A), we can simulate the linear spectra of a confined cavity (Fig.S1C) which reproduced most features from the experimental ones in Fig.S1B. Thus, we confirmed that the doublet peaks that decrease as the IR beam shift outward the trench correspond to the modes in the confined area, while the single peak at higher frequency originates from the cavity outside the trenched area. From the simulation, we observed three more peaks at higher frequencies at 2015cm^{-1}, 2041cm^{-1} and 2071cm^{-1}, which were missing in the experimental data. These peaks were not resolved due to that they were too small and hindered by the background.

Notably, the P mode is missing in both experimental and simulated spectra. The P mode should be between S and D modes. P mode is not a bright mode in our experiment because that P mode is an odd function in space (Fig.S2), and when convoluted with the laser beam mode, the net signal is neglectable. However, when the beam is off-centered, with a smaller beam diameter (~20 µm, 60% smaller compared to experiment condition), and with a high cavity Q value, P modes is resolved (yellow trace in Fig.S2b). The requirements are too strenuous for the current experimental condition, which makes P and other odd modes invisible in our experiment.

To simulate the dispersion curve, we varied the imaginary components of the Gaussian profile, which reflected the plane wave component that is parallel to the cavity as the beam incidence angle changed.

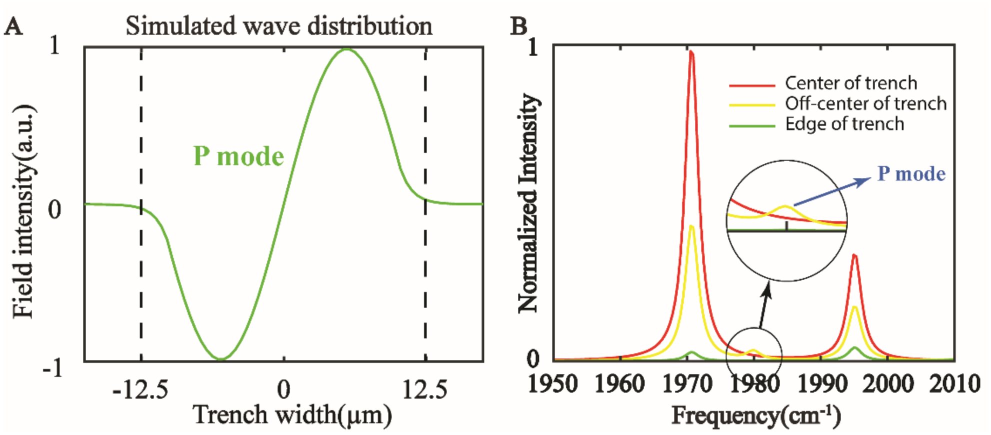

Figure S2 (A) The electric field distribution for P mode. (B) The simulated linear spectra at different positions. To resolve P mode, smaller beam size and higher Q value for the cavity are required. The color indicates different position relationship which followed the convention in Fig.S1.

2.Experimental data for more confined cavities

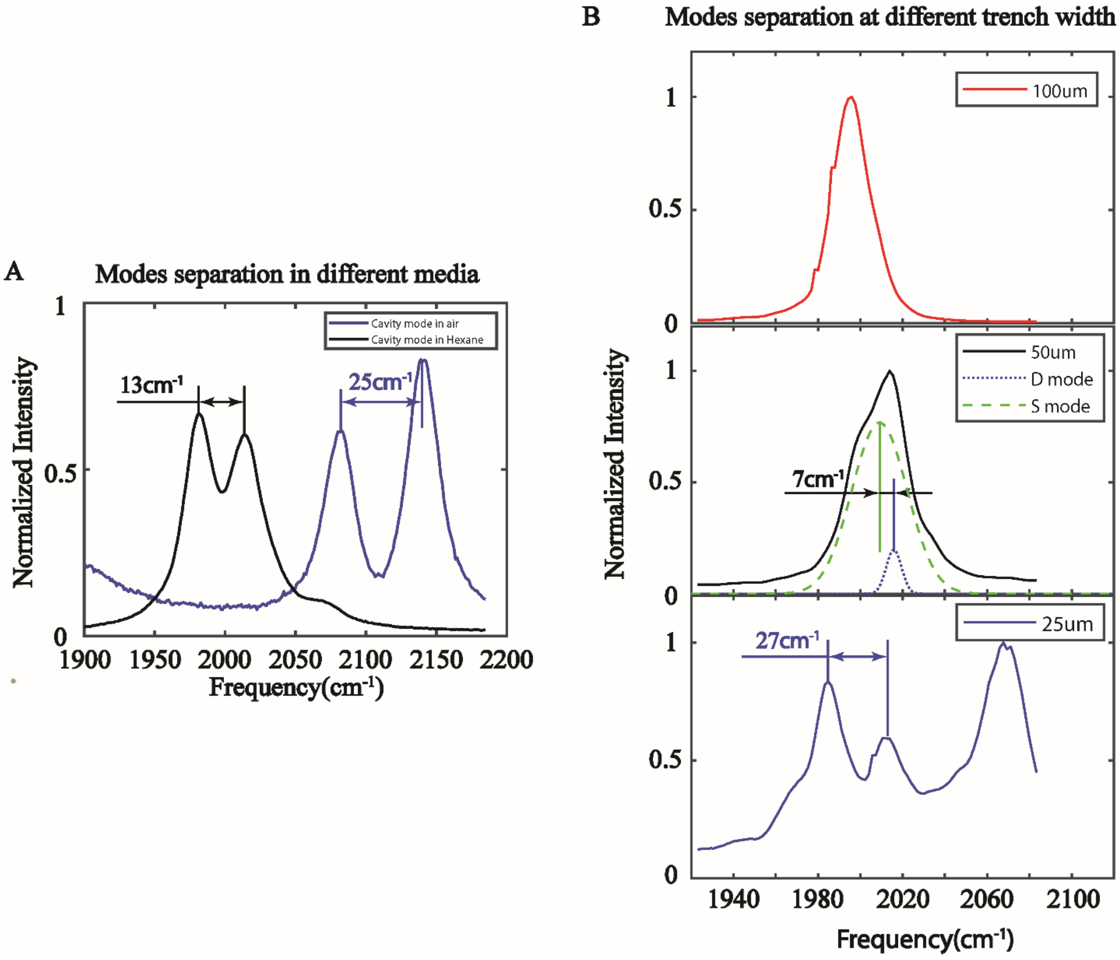

Figure S3 (A) The experimental linear spectra of confined cavity mode in air/hexane (B) The confined cavity mode linear spectrum with different lateral dimension in the air.

The separations between confined cavity modes are functions of media refractive index and the lateral dimension. Specifically, from equation (1), the separation between S and D modes is $\Delta E_{cav} = \frac{\hbar^2 k_{\parallel D}{}^2}{2m_{cav}} - \frac{\hbar^2 k_{\parallel S}{}^2}{2m_{cav}}$. In different media with same lateral dimension (Fig. S3A), the modes separation ratio is

$$\frac{\Delta E_{cav\ in\ hexane}}{\Delta E_{cav\ in\ air}} = \frac{\frac{\hbar^2 k_{\parallel D}{}^2 - \hbar^2 k_{\parallel S}{}^2}{2m_{cav\ in\ hexane}}}{\frac{\hbar^2 k_{\parallel D}{}^2 - \hbar^2 k_{\parallel S}{}^2}{2m_{cav\ in\ air}}} = \frac{m_{cav\ in\ air}}{m_{cav\ in\ hexane}} = \frac{\frac{E_{cav}(k_\parallel=0)}{\frac{c^2}{n_{c\ in\ air}^2}}}{\frac{E_{cav}(k_\parallel=0)}{\frac{c^2}{n_{c\ in\ hexane}^2}}} = \frac{n_{c\ in\ air}^2}{n_{c\ in\ hexane}^2}$$

, where n_c is refractive index of the media inside the cavity. To examine this relationship, we compare the cavity peak separation when the air and hexane were hosted in the cavity. The refractive index of Hexane is 1.375 and air is 1, respectively. This leads to the peak separation ratio to be 1.9 between modes in air and hexane. Experimentally, the peaks separation is 13cm^{-1} in Hexane and 25cm^{-1} in the air (Fig. S3A), agreeing with the model prediction well.

The peak separation between modes in same media is

$$\frac{\Delta E_{cav2}}{\Delta E_{cav1}} = \frac{\frac{\hbar^2 k_{\parallel D2}{}^2 - \hbar^2 k_{\parallel S2}{}^2}{2m_{cav}}}{\frac{\hbar^2 k_{\parallel D1}{}^2 - \hbar^2 k_{\parallel S1}{}^2}{2m_{cav}}} = \frac{k_{\parallel D2}{}^2 - k_{\parallel S2}{}^2}{k_{\parallel D1}{}^2 - k_{\parallel S1}{}^2} = \frac{(\frac{2\pi n_{D2}}{L_{2\parallel}})^2 - (\frac{2\pi n_{S2}}{L_{2\parallel}})^2}{(\frac{2\pi n_{D1}}{L_{1\parallel}})^2 - (\frac{2\pi n_{S1}}{L_{1\parallel}})^2} = \frac{L_{1\parallel}{}^2}{L_{2\parallel}{}^2}$$

,where $L_\parallel$ is the lateral dimension. We tested this relationship by measuring cavity spectra of 100, 50 and 25 μm. At 100 μm (Fig.S3B), only one peak is observed and therefore, the cavity modes did not experience lateral confinement. Two modes started to be resolvable at lateral size of 50 μm. Based on a Gaussian peak fitting, the peak separation was 7cm^{-1}. In contrast, the peaks were well resolved with a lateral size of 25 μm, with a separation of 27 cm^{-1}. Using the relationship at the beginning of this paragraph, the ratio of peak separations between 25 and 50 μm cavities should be ~4, agreeing with the experimental determined value $\frac{27}{7}$ well. We note that this measurement was done using the cavity confined in both lateral dimensions. However, because the in-plane momentum of laser beam was parallel to one of the dimensions, it only probes confinements in one dimension. Thus, the 2D confined cavity followed the same trend and the 1D confined cavities, evidenced by that their cavity mode peak separation remained similar experimentally.

3. Modeling of the cavity thickness dispersion curve with 4x4 Hamiltonian matrix

From the experiment result Fig.3C, we know there should be 4 polariton modes corresponding to 4x4 matrix in the following form:

$$\begin{pmatrix} s & b1 & 0 & 0 \\ b1 & 1983 & 0 & 0 \\ 0 & 0 & d & b2 \\ 0 & 0 & b2 & 1983 \end{pmatrix}$$

Since the polariton modes were formed in solvent hexane, from Fig.S3A we knew in the matrix d-s=13 cm^{-1} (Fig. S3A). In this way, we could solve the coupling strength b1 and b2 at different cavity thickness with the polariton modes position from Fig.3C. We found that b1=b2=21cm^{-1} could optimize the modeled dispersion curve to match with the experimental ones. The modeled dispersion curve was plotted in Fig.3C.

4. 2D IR spectrometer for microcavity system

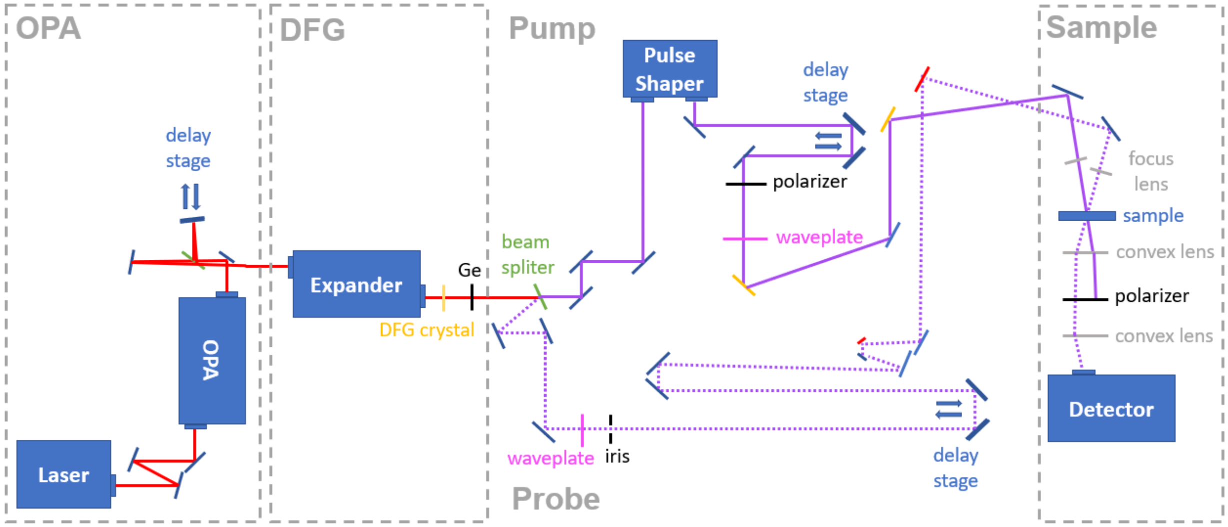

Figure S4 Scheme of two-dimensional infrared experimental setup.

Two-dimensional infrared (2D IR) spectroscopy[1] was applied to characterize the confined cavity polariton system. The setup scheme is shown in Fig. S3. 800-nm laser pulses (~35 fs, ~5 W, 1 kHz) generated by an ultrafast Ti:Sapphire regenerative amplifier (Astrella, Coherent) were sent into an optical parametric amplifier (OPA) (TOPAS, LightConversion) which output tunable frequency near-IR pulses. The near-IR pulses were converted to mid-IR pulses through a difference frequency generation (DFG) process by a type II AgGaS2 crystal (Eksma). After DFG, a CaF_2 wedge split the mid-IR pulse into two parts: the 95% power transmitted part was sent into a Ge-Acoustic Optical Modulator based mid-IR pulse shaper (QuickShape, PhaseTech) and was shaped to double pulses with tunable temporal separation t_1, which formed the pump beam arm; the 5% reflected was the probe beam arm. Both pump (~ 1.1 μJ) and probe (~ 0.2 μJ) were focused by a lens onto the sample.

The pulse sequence is shown in Fig. 3A. Two pump pulses and a probe pulse (pulse duration of 100~150 fs) interacted with samples at delayed times (t_1, t_2, and t_3). After the first IR pulse, a polaritonic coherence was generated, which was converted into a subsequent population or coherence state by the second IR pulse and was characterized by scanning t_1 (0 to 8000 fs with 32 fs steps) using the mid-IR pulse shaper. A rotating frame at f_0 = 1716.3 cm_{-1} was applied to shift the oscillation period to make the scanning step meet the Nyquist frequency requirement. After waiting for t_2, the third IR pulse (probe) impinged on the sample, and the resulting macroscopic polarization emitted an IR signal. The MCT detector (PhaseTech) experimentally Fourier transformed the signal, thus generating a spectrum along the ω_3 axis. Numerical Fourier transform of the signal along the t_1 axis was required to obtain the spectrum along ω_1. The resulting 2D IR spectra were plotted against ω_1 and ω_3. The t_2 time delay was scanned by a computerized delay stage, which was controlled by LabVIEW programs to characterize the dynamic features of the system. A rotational stage was mounted on the sample stage to choose the IR incidence angle and, therefore, the in-plane wavevector of the driven polaritons.

5.Tailoring pump pulse by pulse shaper

In our 2D IR experiments, the pulse shaper was used to turn the incoming transform-limited pulse into a double- pulse with controllable time separation, phase in the time domain, and spectral shape in the frequency domain. Specifically, we set the central frequency and bandwidth of the first and second pulses separately, so that we could control the polariton states being created after each light-matter interaction. For example, we can set the first pulse only centered at UP_p (without exciting other states), and the second pulse to be centered at LP_S, to create a specific coherence state. This method allowed us to create specific coherence without being interfered or overwhelmed by other large signals.

6. Comparison of 2DIR spectra between the dual cavity polariton system and the confined cavity system

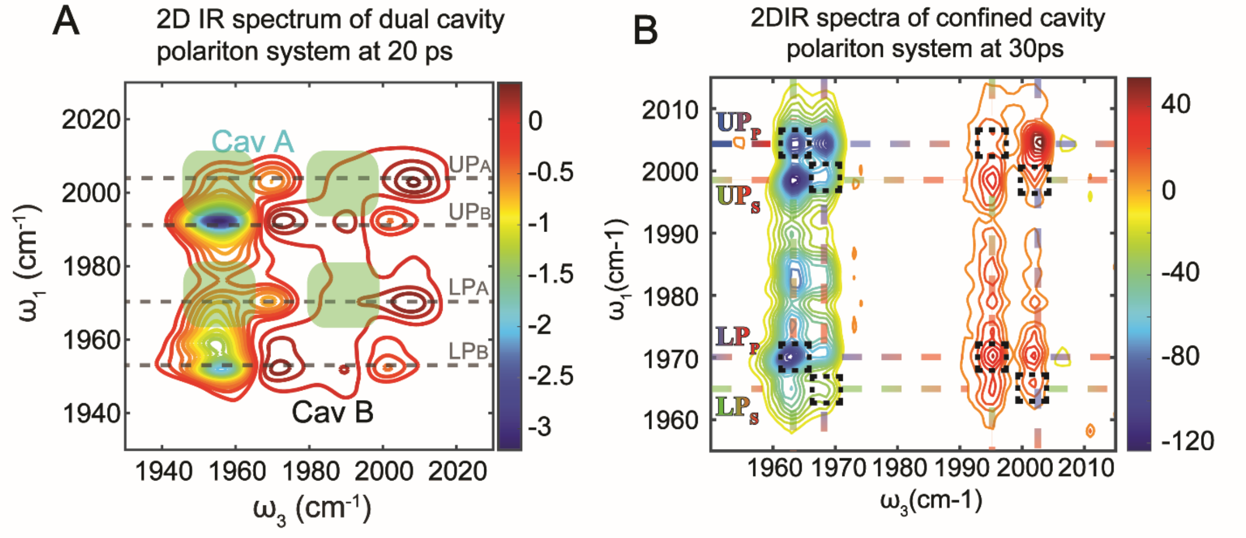

Figure S5 Comparison between the 2DIR spectra of dual cavity polariton system(A) and confined cavity polariton system(B). Comparing to the 2DIR of dual cavity system, the intercavity cross peaks are much more well resolved in confined cavity system (labeled by dash square) which imply that more quantum pathways contributed to those peaks.

7. More arbitrary polariton coherences

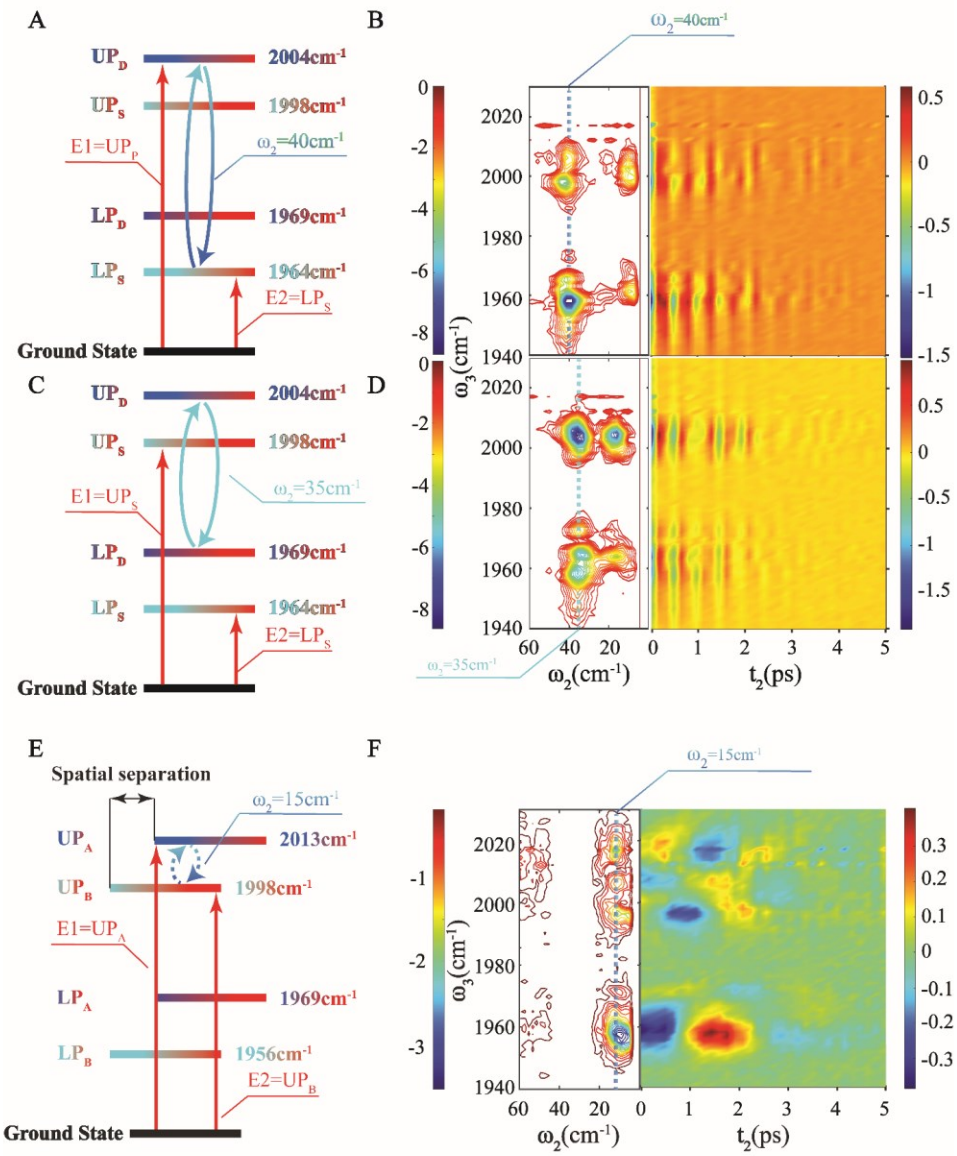

Figure S6 Energy diagram of Rabi oscillation coherence between $|UP_D\rangle\langle LP_S|$ (A), $|UP_D\rangle\langle LP_D|$ (C) and $|UP_A\rangle\langle UP_B|$ (E) corresponding experimental coherence signal along t_2, (B) for $|UP_D\rangle\langle LP_S|$ (D) for $|UP_D\rangle\langle LP_D|$ and (F) $|UP_A\rangle\langle UP_B|$ in frequency domain and time domain respectively.

We tailored the first two IR pulses of 2D IR using pulse shaper to create targeted coherences and scanned t_2 to examine whether any arbitrary coherence could be created. Besides the coherence we showed in Fig.4, we also tailored the first pump pulse to be centered at ω_{UP_P} and the second pulse centered at ω_{LP_S} and ω_{LP_D} individually, to initiate the coherence $|UP_D\rangle\langle LP_S|$ and $|UP_D\rangle\langle LP_D|$ (Fig.S5). Similarly, both coherences were resolved well in frequency and time domain which could support that intercavity coherence was robust in confined cavity polariton system. We note that there were other coherence signals at lower frequencies, which did not match the frequency of the original coherences. Such a signal could be due to coherence transfer, which was out of the scope of this work. We also included coherence $|UP_A\rangle\langle UP_B|$ from previous work[2].

SI References

1. Hamm, P. & Zanni, M. *Concepts and Methods of 2D Infrared Spectroscopy*. *Concepts and Methods of 2D Infrared Spectroscopy* vol. 9781107000 (Cambridge University Press, 2011).

2. Xiang, B., Yang, Z., You, Y. Z. & Xiong, W. Ultrafast Coherence Delocalization in Real Space Simulated by Polaritons. *Adv. Opt. Mater.* **10**, 2102237 (2022).
